# A Tale of Two Twins: Discordant Presentation of COVID-19 in Identical Twins

**DOI:** 10.7759/cureus.25610

**Published:** 2022-06-02

**Authors:** Nicole Chan, Joseph I Berger, Alan Guo, Nirja Inamdar, Mark Samarneh

**Affiliations:** 1 Internal Medicine, St. John’s Riverside Hospital, Yonkers, USA; 2 Neuroscience, Stony Brook University, Stony Brook, USA

**Keywords:** covid-19, nicotine, multiple organ dysfunction, cytokine storm syndrome, twin study

## Abstract

Coronavirus disease 2019 (COVID-19) is a highly contagious viral illness caused by the RNA virus Coronaviridae subtype severe acute respiratory syndrome coronavirus 2 (SARS-CoV-2). Rapid infection caused by this virus became overwhelming and resulted in millions of deaths worldwide. The effects of smoking have been heavily studied and lead to increased occurrence of COVID-19 viral infections and mortality. The phenomenon of cytokine storm has been shown as one of the leading factors of mortality. However, the question remains as to what factors, either genetic or environmental, ultimately lead to the increased incidence of cytokine storms compared to others.

We present a case of two cohabitating, 57-year-old, male, identical twins (Twin A and Twin B) who contracted SARS-CoV-2 simultaneously. Both Twin A and Twin B had similar medical histories, except for Twin A being a former smoker while Twin B a current smoker. While both twins presented with cough and shortness of breath, Twin A also presented with hypoxia, leukocytosis, evidence of acute kidney injury, and transaminitis while Twin B presented normoxic with solely tachycardia. Due to his presentation and vital signs, Twin B received Bamlanivimab but developed hypoxia during the infusion. Both twins were subsequently initiated on Remdesivir, dexamethasone, and supplemental oxygen daily. After completion of treatment courses, both twins had improvement in their laboratory values and were subsequently discharged with supplemental oxygen to be further weaned in the outpatient setting. Due to the twins’ cohabitation, contracting SARS-CoV-2 simultaneously, and similar medical history, we highlight the potential mechanism of nicotine’s chemical ability to blunt the subsequent inflammatory process of COVID-19.

Despite nicotine’s potential ability to dampen cytokine storms, smoking has well-documented adverse effects and we, like many experts, entirely discourage it. However, given the rare situation of identical twins contracting SARS-CoV-2, we can extrapolate information regarding the effects of the virus without obfuscation from genetic and environmental factors to identify areas of research for new therapies.

## Introduction

Coronavirus disease 2019 (COVID-19) is a highly contagious viral illness caused by the RNA virus, Coronaviridae, specifically, subtype severe acute respiratory syndrome coronavirus 2 (SARS-CoV-2). SARS-CoV-2 was initially discovered in Wuhan, Hubei Province, China, in late December 2019 presenting primarily as a respiratory viral illness. It quickly propagated across the globe resulting in prolonged shutdowns, disruptions in both micro and macroeconomics, and massive mortality. Additionally, healthcare systems worldwide have been overwhelmed, limiting their capabilities in controlling the spread of the disease and ultimately resulting in over 5,000,000 deaths worldwide [1]. Due to its deadly global impact, it is imperative to identify the pathophysiology of the virus and develop new therapies.

We present a case of identical twins who contracted COVID-19 simultaneously. Together with this case, there are currently three known instances of simultaneous contraction of COVID-19 by identical twins in the United States [2,3]. Simultaneous evaluation of this case allowed for a side-by-side comparison of morbidity and mortality while removing the effects of genetic effects due to their shared DNA.

## Case presentation

We present the intriguing case of two cohabitating 57-year-old males, identical twins (hereinafter referred to as Twin A and Twin B), with a medical history of class III obesity, hypertension, hyperlipidemia, and obstructive sleep apnea who presented to the emergency room with cough, shortness of breath, and loss of smell of one-week duration after exposure to their COVID-19-positive family member. Both twins were unvaccinated for COVID-19 prior to their exposure. Subsequent laboratory testing revealed that they were both COVID-19 positive as well as antibody positive.

While the initial presentation was equivocal, Twin A and Twin B differed in their social history and disease course. Notably, Twin A had a former smoking history, now resolved, except for three cigars per year, with his last cigar six months ago. Twin B was a current smoker having smoked 40 cigars in the past four months, with a recent increase in usage. Both twins had labor-intensive occupations and exercised routinely. Twin A worked as a landscaper with an exercise regimen of using a stationary bike four times a week at 20-minute intervals. Twin B was a former firefighter, a former soccer player, and used a stationary bike daily at 45-minute intervals.

Case 1: Twin A

**Figure 1:**
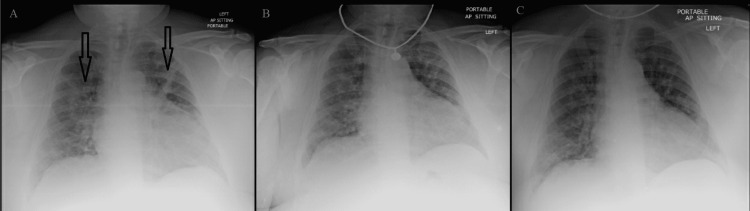
Twin A: chest X-ray on day one (A), day three (B), and day five (C). Arrows depict patchy bilateral infiltrates.

Twin A presented with physical examination findings of coarse breath sounds heard bilaterally and vitals showing hypoxia at 90% oxygen on room air, with other vitals remaining stable. Laboratory findings were significant for an elevated hematocrit, leukocytosis, elevated creatinine, transaminitis, and inflammatory markers (Table 1). Chest X-ray was significant for patchy infiltrates present in bilateral lung fields (Figure 1). Prompt treatment for COVID-19 was started with a five-day course of Remdesivir, empiric ceftriaxone (two doses), azithromycin (one dose), vitamin C and D, zinc, and dexamethasone 6 mg/day. Empiric ceftriaxone and azithromycin were started due to concern for superimposed community-acquired bacterial pneumonia. The acute kidney injury was treated with fluids and subsequently resolved. Transaminitis also improved after day four. Following completion of Remdesivir, the patient completed a pre- and post-oxygen test with a resting oxygen saturation of 91% without supplemental oxygen and a subsequent drop in oxygen saturation to 85% while exercising. His saturation improved to 92% after supplemental oxygen of 2 L indicating a requirement for home oxygen and was subsequently discharged in stable condition on day six.

**Table 1:** Twin A: laboratory trends of liver enzymes, renal function, and inflammatory markers

Laboratory name	Day of admission	Day three	Day five	Reference range
Aspartate aminotransferase	49	57	44	15–37 U/L
Alanine aminotransferase	76	112	187	13–61 U/L
Creatinine	1.4	1.2	1.2	0.55–1.3 mg/dL
D-dimer	1,604	1,219	1,055	0–500 ng/mL
Ferritin	461.7	520.7	400.8	8–388 ng/mL
Lactic acid dehydrogenase	425	311	254	87–246 U/L
C-reactive protein	7.7	2.2	0.7	0.0–0.3 mg/dL

Case 2: Twin B

**Figure 2:**
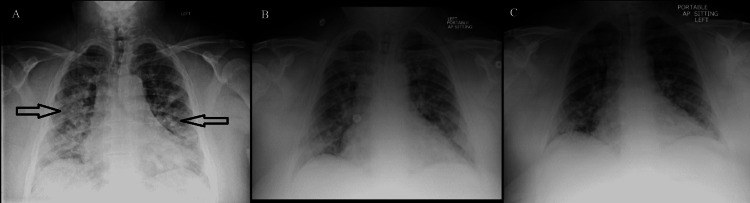
Twin B: chest X-ray on day one (A), day three (B), and day five (C). Arrows depict diffuse bilateral infiltrates.

Twin B presented with physical examination findings of coarse breath sounds heard bilaterally, with vitals showing 95% oxygen saturation on room air and tachycardia of 120. Laboratory findings were only significant for elevated inflammatory markers (Table 2). Chest X-ray was significant for diffuse bilateral infiltrates in bilateral lung fields (Figure 2). Due to his vitals on admission, he was initially treated with Bamlamnivimab; however, two hours following the infusion, he desaturated to 92% on room air. Subsequently, treatment with a five-day course of Remdesivir, vitamin C and D, zinc, and dexamethasone 6 mg/day was started. He was also given ceftriaxone and azithromycin empirically for possible superimposed community-acquired bacterial pneumonia. Following the completion of Remdesivir, the patient completed a pre- and post-oxygen test with 95% oxygen saturation on room air at rest, 85% with ambulation on room air, and improvement to 94% with 2 L of supplemental oxygen. This indicated a requirement for home oxygen, and he was subsequently discharged in stable condition on day six.

**Table 2:** Twin B: laboratory trends of liver enzymes, renal function, and inflammatory markers.

Laboratory name	Day of admission	Day three	Day five	Reference range
Aspartate aminotransferase	34	39	24	15–37 U/L
Alanine aminotransferase	43	94	71	13–61 U/L
Creatinine	1.2	1.2	1.1	0.55–1.3 mg/dL
D-dimer	1,355	1,143	803	0–500 ng/mL
Ferritin	367.0	360.8	191.4	8–388 ng/mL
Lactic acid dehydrogenase	404	325	468	87–246 U/L
C-reactive protein	7.8	3.3	1.0	0.0–0.3 mg/dL

## Discussion

In our case, Twin A presented with hypoxia, leukocytosis, elevated creatine, and transaminitis while Twin B presented normoxic with solely tachycardia. The only major divergent characteristic remains Twin B’s infusion of Bamlanivimab and his nicotine usage. It has been noted that Bamlanivimab presents a notable difference in treatment; however, laboratory differences were present prior to infusion, suggesting that the aberrancies existed before therapy and were likely a result of coronavirus and its pathological process.

The entry of SARS-CoV-2 involves the host receptor angiotensin-converting enzyme 2 (ACE2), which is present in both the lungs and liver parenchyma [4], as well as the cellular protease, transmembrane protease, serine 2 (TMPRSS2). It has been proposed that certain genetic factors, such as polymorphisms in the *ACE2* and *TMPRSS2* genes [5], along with differences in ABO blood groups or polymorphisms in the human leukocyte antigen class I genes, play a role in the differences in disease severity of SARS-CoV-2 [6]. However, due to our patients being identical twins and thus identical genetic makeup, there are no significant polymorphisms in regards to the entry of the virus. Twin A had significantly different laboratory findings than Twin B, suggesting that the disease course is affected by more than genetics alone. We posit that this difference can be attributed to social factors, including smoking history and nicotine exposure, as well as the novel features of SARS-CoV-2 itself. Smoking tobacco has been clearly demonstrated to increase the risk of respiratory infections [7]; however, much knowledge can be gained from investigating its effect on the nicotinic cholinergic system and the impact this has on COVID-19 infection to ultimately design new therapies.

Cytokine storm is a major cause of mortality in COVID-19, and many current treatments are directed toward inhibiting it [8-10]. The nicotinic cholinergic system, which mostly involves a7-nicotinic acetylcholine receptors (a7-nAChRs), participates in anti-inflammatory pathways, and its disruption by the virus has been reported to prevent an immune response; this prevents the binding of endogenous acetylcholine and leads to the subsequent cytokine storm response [11]. Interestingly, activation of the nicotinic cholinergic system has a profound effect on reducing the activity of the immune system. Although the binding of nicotine to a7-nAChRs leads to upregulation of ACE2 receptors and thus increases COVID-19 transmission, stimulation of a7-nAChRs by nicotine has also been documented to block pro-inflammatory cytokines, including tumor necrosis factor-alpha, interleukin (IL)-1, and IL-6, thus blocking the impending cytokine storm. SARS-CoV-2 S1 protein and its binding to the nAChR cause the dysregulation of the nicotinic cholinergic system. Smoking behavior has also been associated with decreased natural killer cells and cytotoxic T lymphocytes, both of which are imperative to combat virus-infected cells, leading to another mechanism in the suppression of immune response [12,13]. Together with our presented case, these investigations reveal that targeting nAChRs to blunt the ensuing cytokine storm may result in better COVID-19 patient outcomes.

In this study, Twin A was a former smoker while Twin B was a current smoker. Although smoking causes an increased incidence of liver disease, Twin A unpredictably developed liver disease despite his former smoker status. Studies have shown that hemoglobin and hematocrit levels in smokers, compared to non-smokers, were increased in statistically significant amounts. This has been theorized as a response to hypoxic peripheral stimulation leading to a compensatory increase in hematocrit and hemoglobin to maintain oxygenation [14]. Studies involving high-altitude acclimatization between smokers and non-smokers further revealed that smokers were less likely to develop acute mountain sickness. They were less likely to develop headaches, anorexia, nausea, vomiting, and sleep disturbance that results from hypoxia-induced cerebral vasodilatation [9]. In addition to this acclimatization, there is the phenomenon of decreased inflammation associated with smoking. Examples of his effect are illustrated by smoking’s defensive association with ulcerative colitis; however, the mechanism is not fully understood [15]. It is noted that smoking is an all-cause risk factor irrespective of the disease and that the potential anti-inflammatory effects of pharmacologic nicotine must be balanced with the increased risk of COVID-19 entry via receptor upregulation [16]. Despite these instances of defensive mechanisms and the body’s ability to adapt to hypoxic environments involved in smoking, hypoxia is detrimental to various organ systems, especially those that are highly vascularized and require optimal oxygenation for proper functioning.

The mechanism by which COVID-19 causes liver injury is still being debated; however, there are several proposed mechanisms, including direct liver injury, drug-induced liver injury, aggravation of the underlying liver disease, hypoxia-associated liver damage, and cytokine storm [17,18]. The act of smoking has also been found to contribute to liver injury through direct toxic effects, impaired oxygen transport, and resultant tissue hypoxia [19]. Although Twin B is an active smoker, he did not have the expected liver injury, demonstrated by his laboratory values on admission. Our patients both demonstrated similar levels of total bilirubin making direct damage less likely and were both exposed to the same levels of Remdesivir, accounting for its hepatotoxic effects. Neither had evidence of underlying liver disease. Hence, we propose that the damage was related to the cytokine storm phenomenon as well as hypoxia-induced liver injury.

## Conclusions

Twin A was a former smoker but presented with new transaminitis and acute kidney injury after his infection with SARS-CoV-2. Whereas Twin B was a current smoker, presented normoxic, and did not have multiorgan involvement. We suspect that the difference in smoking history and increased serum nicotine in Twin B may have played a part in blunting the inflammatory process of COVID-19. Despite this factor, smoking is associated with higher rates of hospitalization, risk of infection, and mortality. However, with this information, we hope to shed light on an area of investigation for treatment as the world continues to combat this disease. With this case, we hope to introduce another potential target of treatment relating to nicotine and its receptors. As there is very limited data on cohabitating, identical twins, contracting COVID-19 simultaneously, and being monitored in the same facility, we firmly believe that more research needs to be done.
